# Reviews

**Published:** 1896-01

**Authors:** 


### REVIEWS.

*The Dog in Health and Disease.* By Prof. Wesley Mills, McGill University. Second edition; published by D. Appleton & Co., New York. So rarely has the successful sale of books of special interest to the veterinary profession demanded a second edition, that it must prove very gratifying to the author of this valuable work to enjoy such an experience, and in so short a period of time. Many new points have been added to the book, and much valuable information added bearing upon medication, that we are sure that many of those possessing a copy of the first edition will want one of the second. Prof. Mills makes a good point in his assertion that those who are to give succor to the dog in disease should be fully acquainted with the dog in health, and familiar with the various breeds and types of dogs, to all of which we give hearty approval.

The American and Continental Sanitas Co. has just issued, in connection with its many preparations, a small book entitled *How to Disinfect.* To those who have been using Sanitas preparations it will prove a handy and convenient reference and guide as to the most effective preparations in the hospital, stable, kennel, poultry-house, etc.

*Practical Toxicology.* For Physicians and Students. By Prof. Dr. Rudolf Kobart. An authorized edition of this work, translated by L. H. Friedburg, Ph.D., Professor of Chemistry and Toxicology at the American Veterinary College, is in press, by the publishing house of W. R. Jenkins, New York City.

